# Remarks on Pelvic Inflammations, and the Management of Their Residues

**Published:** 1888-07

**Authors:** William Warren Potter

**Affiliations:** Buffalo, N. Y.; 284 Franklin Street


THE BUFFALO Medical AND Surgical Journal

VOL. XXVII. JULY, 1888. NO. 12.

### Original Communications.

### *REMARKS ON PELVIC INFLAMMATIONS, AND THE MANAGEMENT OF THEIR RESIDUES.*

BY WILLIAM WARREN POTTER, M.D., Buffalo, N.Y.

The habit that formerly prevailed, sanctioned to a certain extent by custom, of characterizing almost any inflammation in the pelvis, without regard to its origin or involvement, as pelvic cellulitis, was misleading as well as incorrect, and it is, therefore, proper that it should give way to the term, pelvic inflammation, as being a more comprehensive and better expression every way. It would seem a good plan to group all inflammations of the pelvic structures under the general designation of pelvic inflammations, and to then differentiate them, if possible, according to their origin or location, when discussing the special diseases of the organs and tissues in severality.

The question of differentiation as to origin is one fraught with much difficulty, and is likely to remain undetermined as long as acute and conscientious observers hold conflicting views regarding the initial or primitive pathological conditions, found lying at the very threshold of the subject.

Perhaps the meaning I desire to convey will be better understood if I illustrate: One group, and a very large one, I may add, with Dr. Emmet at its head, believes that the majority of these inflammations begin in the cellular tissue, and involve other organs and structures, if at all, by extension therefrom. Dr. Battey and his followers hold that the serious inflammations which are so destructive in their consequences, depend primarily upon disease of the ovaries, reaching other structures from that starting-point. Still another class of observers, whose numbers appear to be constantly increasing, in which Dr. Gill Wylie may be mentioned as a conspicuous leader, attribute these inflammations to diseased tubes as originating points of departure. I would not affirm that these distinguished men are engaged in acrimonious antagonism relative to the origin of pelvic inflammations, for, as a matter of fact, there is much common ground of agreement between them upon many points which the subject involves; nor would I wish to be understood to quote them as having pronounced a final dictum upon the point in question; but simply that this is, succinctly, the trend of their opinions thereupon at present, or up to the latest expression thereof that I have observed. Though purposely limiting my references in this illustration to gynecologists in this country, it may be remarked that these several views are supported by distinguished foreigners. I have no doubt all of them are correct; for, unless I have very much mistaken the order of sequence in the clinical phenomena that I myself have observed, there are inflammations that begin in the connective tissue surrounding the uterus, and are conveyed to other points by the blood-vessels or lymphatics, or have extended into them through continuity or contiguity; and, again, there are inflammations that originate in the ovary, either as a simple ovaritis or as cystic or cirrhotic disease, and have traveled both upwards and downwards in their course until there has been a general pelvic cellulitis or peritonitis, or both combined; and, yet again, that there are other inflammations which have started in the tubes themselves as a center, and have reached out into adjacent or contiguous structures, or have been carried hither and yon through the medium of the absorbents.

The question of the importance of pelvic inflammations I should like to say a word upon, since I have observed that it has been challenged in certain quarters, so far, at any rate, as "minor pelvic inflammations"—whatever that may mean—are concerned. Now, to my mind, no inflammation, whatsoever be its origin or limitation, should be looked upon as, in any sense, a "minor" malady, or one whose importance is likely to be "exaggerated," which has once invaded the female pelvis. It may be that their anatomical residues, as ascertained in the post-mortem room, appear somewhat trifling or unimportant in some instances. Perhaps, the complete clinical histories of these very cases, could they be known, would throw additional light upon the importance of the inflammations that left these traces or remnants behind. The debris along the shore does not always indicate, nor with great certainty, the real size of the wreck, much less surely the violence of the storm, which may have caused the disaster. I have been much interested in Dr. Coe's paper on this subject, (*N. Y. Medical Journal*, May 15, 1886,) and particularly in what he says bearing on the propriety of doing minor operations in the genital tract, when these inflammatory residues are found present. It is quite possible that, in certain cases, such operations have been done with impunity, or without resultant harm; but I have always been accustomed to regard the remnants of old inflammations in the light of danger signals, and I find it difficult to rid myself of such impressions.

Permit me to remark just here, that it is no part of my purpose to make this brief paper controversial, nor to do injustice to anyone. I am merely summoning a few of the witnesses for a re-examination, with a view, if possible, to arrive at the truth concerning some doubtful points, and to ascertain what can be done towards clearing them up.

Whatsoever the origin of these inflammations may be, the question of their clinical importance need not engage our attention very long; for I take it as a granted fact, that the various phenomena now generally grouped under the name of "pelvic inflammations," play an important part in the etiology of by far the largest proportion of the disorders for which the gynecologist is consulted. It may be that they take their start in septic infection from gonorrhœa, traumatism, or parturition; or, on the other hand, their nidus may be the very pink of benignity itself; they may begin in the cellular tissue, in the peritoneum, or in the endometrium; as a vaginitis, metritis, salpingitis, or lymphangitis; it matters not when nor where nor how they start, they are inflammations pure and simple in their beginning, varying in degree of intensity and in resultant complications according to the constitution of the patient, virulence of the infection, benignity, or organ involved.

It is probable, as I have already hinted, that they sometimes begin in the pelvic connective tissue surrounding the uterus, at other times in the peritoneum, and at still others in the tubes themselves. They sometimes proceed promptly to resolution; again the masses of exudation will remain unaltered for months or years; yet again they go on to abscesses, and still yet again to other formidable and serious mischief, either in the site of their origin, or, by extension to other tissues and organs, ravage the pelvis to an extreme degree. In the septic varieties, the infection may be carried through the medium of the blood-vessels directly, or through the lymph-channels; but, come how they will and travel how they may, they are an ever-present, all-sufficient source of suffering and ill-health; and, moreover, often lead to important complications and sequilæ. In a certain number of cases, the inflammations will probably remain within a circumscribed limit, involving only the connective tissue adjacent to, or surrounding, the uterus in the sub-peritoneal pelvis. The exudate in these cases, under appropriate treatment, becomes absorbed, and very little or no permanent harm ensues. In others, however, the septic material is carried by the blood-vessels or lymphatics, or extends by continuity or contiguity, as the case may be, beyond the boundaries of the cellular tissue, the peritoneum is invaded, the uterine appendages becoming, perhaps, deeply involved in the inflammatory processes, and more serious mischief follows.

Thus it would appear that we are liable to meet with several varieties of pelvic inflammations, and, therefore, for the sake of convenience as well as for greater simplicity in description, I would suggest the following classification :

1. Simple pelvic inflammation. (Pelvic cellulitis.)
2. Compound pelvic inflammation. (Cellulo-peritonitis.)
3. Complicated pelvic inflammation.

In the first order, I would group all the simpler varieties of pelvic inflammation, that merely involve the circum-uterine connective tissue, not extending above the sub-peritoneal pelvis. The second could be made to embrace those inflammations that reach into and include the pelvic peritoneum in their progress, more formidable than the first, and are of a cellulo-peritoneal character. The third proposed classification would naturally infold those forms that involve the appendages, such as depend upon the specific infection, and those that end in pus formations or have other grave terminations. This arrangement of the subject would enable us to avoid the use of those objectional and misleading terms, peri- and parametritis.

Dr. Emmet has taught us more about the importance of pelvic cellulitis,—the necessity of its early recognition, its relations to other diseases of the sexual organs, and its appropriate treatment,—than any other observer ; and the profession can never fail to give him the first place in its affectionate regard for the thoroughness, clearness, and force, in which he has insisted upon the necessity of heeding the lessons that he has taught. They are stamped with ineffaceable colorings upon the whole system of modern gynecology. But, in view of the continual enlargement of the field of knowledge generally, and particularly since tubal pathology has become better understood, it is probable that these earlier lessons have outgrown their original proportions and significance; certainly they are reaching out in a wider scope to-day than the great master could himself have foreseen when he first announced his doctrine of pelvic cellular pathology.

Around the modern doctrines of tubal disease, which have chiefly come into vogue within a decade, are gathering a large number of younger gynecologists who are rather loth to adopt any views of pathology that seem to counterpoise the theory of the origin of these inflammations elsewhere than in the appendages; while the older observers in this field are equally slow, as a rule, to yield ground that experience would seem to strongly fortify. It must be confessed that these newer views have served to embarrass the defense of the former positions held by the profession on many points connected with the pathology of pelvic disease, and a recasting of the lines may become necessary before a true solution is reached. Be that as it may, the real object of all scientific research in medicine is to establish facts, and these without regard to the theory of any man, or coterie, or any priority of discovery, or the particular claims of anybody to any operation, method, or special mode of treatment whatever. These are only interlocutory questions that chiefly concern the historian.

The medical axiom that proper treatment must, of necessity, be based upon accurate diagnosis; and its corollary, that diagnosis must, in turn, rest upon correct pathological knowledge, is as applicable here as elsewhere. Nevertheless, owing in part to the difficulties surrounding their investigation, and in part to the disguises in which the disease frequently masquerades, it is not always possible to mark the diagnostic line sharply between a cellulitis and peritonitis, nor between an ovaritis and a salpingitis; especially so when these several conditions blend and intermingle in their progress, as not seldom happens. How many operators are able to satisfy even themselves in advance as to what will be found when they open the abdomen? Even Mr. Tait, if we do not entirely misapprehend him, boldly asserts that when in grave doubt about the diagnosis, and the symptoms are of a serious or threatening nature, he lays open the abdomen to settle the matter.

This is a cavity full of surprises—a cavum consternatum, so to speak—and he is often the most surprised whose diagnosis is varified by laparotomy. Those most experienced in the operation are often slowest to announce a positive diagnosis, hence it is getting to be considered nigh unto an index of skill to give, before section, a conservative opinion as to the anatomical characters of the abdominal and pelvic contents.

These inflammations begin in so many different ways, that it is not strange they become shrouded in considerable mystery in their advanced stages, or after they have passed beyond the confines of the structures originally involved. Thus it becomes more difficult to diagnosticate differentially, when unfamiliar with their inception and early progress. Let me illustrate, or apply, my meaning by what a lawyer would term a hypothetical case: Suppose an inflammation of the pelvic connective tissue to begin, for instance, in its parietal layers along the uterus, thence invading the loose areolar tissue around the vagina and cervix, finally extending to the broad ligaments, and the sheaths of the blood-vessels and lymphatics. The peritoneum now becomes involved, a pseudo-membrane is formed which, by its contraction, displaces the pelvic organs, consolidates and binds them together, matting uterus and appendages together into a tumor-like mass. Suppose, further, that such a case, after the lapse of time and the exhaustion of patience in vainly hoping for recovery, finally seeks the advice of the gynecologist, who finds himself suddenly called upon to decide its nature and the methods to be adopted for its cure. Would not his task be much simplified, and his judgment greatly aided, by a previous knowledge of the case from the inception of the inflammation to its termination? And this brings me to remark, that the gynecologist rarely sees these inflammations in their first forms. They usually appear as acute or subacute inflammations, finally becoming chronic; the acute and subacute forms generally fall to the care of the family physician, so the gynecologists, seeing them chiefly in their chronic conditions, deals mainly with their remnants or residues.

If the initial inflammations appear in a variety of forms and progress through many avenues, what shall be said of the multifarious aspects that their remnants present? If the former are perplexing in their behavior, the latter are treacherous, hidden, even deceptive, and their name is almost legion. They are manifested in altered functions, changed structure, and perverted sensation in all the organs and tissues of the region invaded, and are a constant menace to the comfort, safety and fruitfulness, not to say physiological perfection, of women so afflicted. They spring chiefly from three sources: 1st, abortion and parturition; 2d, traumatism; and 3d, infection.

The list of these remnants that may be the result, directly or indirectly, of parturition alone is a long one; but I will not take time to even enumerate them now. I merely wish to speak of one or two varieties. It is well known that in exudative cellulo-peritonitis, masses of the exudate frequently become encapsulated and remain unchanged for a long time; finally, something happens to light up the inflammation anew, and changes then take place in a variety of ways. The commonest are: (1) resolution and absorption of the exudation; (2) pus may form and the abscesses open into the rectum, vagina—rarely into the bladder—and occasionally into the peritoneal cavity. I have lately seen an example of each of these modes of termination of residues after parturition. In one, several months after labor, a large tumor-like mass was found in the retro-uterine space, which, though quite firm and hard, was believed to contain pus. An exploratory puncture proved this suspicion correct, a free incision and drainage through the vagina were employed, and the case went rapidly onward to recovery. In another case, the mass was somewhat boggy, quite large, caused impaction of the pelvic viscera, and numerous reflexes—stomachal and psychical. Absorption finally took place, and this patient made a good recovery. These cases both, but especially the latter, resembled retro-uterine fibromata, and had been mistaken for tumors of that sort—a not unlikely error in this kind of inflammation residues.

Other important remnants or residues of pelvic inflammations are: Cicatrices and hypertrophic enlargements of the cervix, due to lacerations; fixation of the womb, with or without displacements; subinvolution; matting together of uterus, tubes and ovaries by pseudo-membranes; cystic, cirrhotic, hemorrhagic, and pyogenic ovaries; pyo- hydro- or hematosalpinx, resultant from catarrhal or gonorrheal salpingitis; and cicatrices of the uterine supports.

The influence of the residues of pelvic inflammations upon the health of women who chance to possess them, varies in degree and importance according to age, constitution, resisting power, and extent and character of the invasion; ranging all the way from slight or no disturbance whatever, to severe and even dangerous manifestations of the gravest organic disease. Again, they may cause very little inconvenience at first; but, secondarily, as time advances and complications arise, may occasion the most serious threatenings to life. Recurrent inflammatory attacks may be noted as especially taxing to the stamina of the female. After an apparent recovery and everything promises ultimate sound health, the attack breaks out again, perhaps on the other side, going through all its phases, and finally stranding the patient on the shoals of sterility, or chronic invalidism. Frequently, too, the remote or sympathetic disturbances to the economy which these residues provoke, are less endurable than the primary disease. The rectum, bladder, and stomach are particularly liable to functional disorders of a distressing and misleading nature. Digestive ailments and skin diseases are now and then attributable to these residues. Finally, reflex and sympathetic neuroses, and psychic disturbances, are often a distressing and obstinate accompaniment, ranging all the way along the line from mild hysteria to absolute insanity.

The list might be further extended, as well as elaborated in greater detail, but enough has been said on this point to, at least, emphasize the importance of early recognition and prompt attention to even the lesser products of these inflammations.

### MANAGEMENT OF INFLAMMATORY RESIDUES.

The management of pelvic inflammations naturally divides itself into that for the acute or immediate stage, and that for the chronic or remote form—the latter embracing the treatment of the residues or remnants of the inflammatory processes. The therapy of acute inflammations is not considered as within the scope of this paper; nevertheless, it may be remarked that it is important to prevent or modify the attacks, as far as possible, by the thorough use of antiseptics during labor or after delivery. Without going into this subject in detail, it may be laid down as essential that the hands of the accoucheur should be absolutely sterilized to begin with; that in instrumental labors, or in any case where it has been necessary to invade the uterus either with hands or instruments to effect delivery, the womb should be irrigated with an antiseptic liquid that is an undoubted germicide; and that this process should be repeated in any such case where the temperature rises above 100 F., with a tendency to steadily increase, sufficiently often to keep it free from sepsis. Under proper antiseptic precautions in midwifery, pelvic inflammations will often be prevented, or their ravages diminished.

When a woman does not effect a "good getting up" after delivery, and she complains of deep-seated pelvic pains, especially if associated with a slight rise in temperature which continues for days or weeks, the suspicion is very strong that there are exudations lurking somewhere in the pelvic cavity, for which a careful search should be made. If they should be discovered, appropriate treatment, with a view to promote their absorption, should be instituted at once. In such cases, I have usually obtained benefit from a careful tamponnement of the vagina, medicating the tampons according to the nature of the case. This furnishes support, rest to the parts, and elastic pressure, all of which greatly facilitate absorption of the exudate, and tend to relieve pain. It is a valuable adjunct in the management of all the residues of pelvic inflammations in the preparatory or initial stage of treatment, and sometimes seems to effect a complete cure, rendering the appeal to more radical measures unnecessary.

In a paper read before the Medical Society of the State of New York, a few years ago, I advocated the use of the vaginal multiple tampon in the treatment of uterine and ovarian displacements. The tampons, at that time, were made of cotton, generally medicated in some way, and always disinfected. In these pelvic inflammations, I prefer wool, as furnishing equally good support, and much better elastic pressure, which is a point of great importance. Finely carded lamb's wool is soft and unirritating, and is much more useful in these cases than the ordinary cotton tampon. The principle of elevating the uterus and its appendages out of the lower pelvis, releasing them to a considerable extent from impaction, and thereby promoting nutritive changes, is undoubtedly a correct one; but its usefulness is restricted within certain limitations. Its contra-indications appear to be fever, sub-acute or chronic peritonitis, strong adhesions, and abscesses. Its use should never be persisted in when it causes pain, but when it affords relief, its employment is pretty certain to be followed by good results. In doubtful cases, it would be well to proceed cautiously at first, until its efficacy is tested, after which, if favorably indicated, we may go ahead with confidence.

Under the stimulus of the elastic pressure of the wool tampon, and the support it affords, the exudates that have been thrown out during the acute inflammation, and which render the tissues sodden and boggy, are frequently absorbed, the circulation in the blood-vessels and lymphatics becoming freer, and the nutrition, as a consequence, becomes improved. Even in fixation of the womb by adhesions, when the latter are not too strong, it is possible to loosen the organ, free it from its moorings, and restore it to, at least, an approximative normal position through this method of support and pressure. In subinvolution, too, the essential shrinkage of the uterus can often be promoted by this means.

The residues due to laceration of the cervix or other portions of the utero-vaginal tract usually require surgical measures for their cure. It appears, to the writer, that one of the important benefits of trachelorrhaphy, among its many others, is found in its prevention of, or the relief it affords from, those secondary conditions liable to follow in the train of an unrepaired cervical rent—either tissue changes, reflex neuroses, or an extension of the inflammatory products into other organs or tissues. Hence, in making the operation, it is expedient to excise all cicatricial tissue and hypertrophic thickening of the everted cervix.

In displaced uteri, caused by inflammatory remnants, a reasonable effort to free the organs from their moorings, and restore them to their proper positions, may be made, but I need scarcely remark that it should not be attempted with the uterine sound or other intra-uterine redresser. The sound has little or no place in the management of these cases, either for purposes of diagnosis or treatment; indeed, it may be confidently asserted that it is a fruitful source of mischief, liable to rekindle a latent inflammation, and to even provoke new complications. Pessaries, too, are of no avail, and may do positive harm, if resorted to before the uterus is made to move freely in the pelvic cavity. If this mobility can be re-established through any agency free from harm or danger, then a suitable pessary may prove serviceable.

The formation of pus in the pelvic cavity is not an infrequent ending to either acute or chronic inflammation, and, when established, furnishes an interesting field for the surgeon. When large abscesses form as the result of acute inflammations, it is not difficult to decide upon the proper course to pursue; but such is not always the case when old residues finally break down into pus sacs. In the former, the symptoms are urgent, something must be done, and that quickly, or disaster may result. Moreover, the location of the abscess is not, as a general rule, difficult to define, nor the pus itself out of easy reach. Not so, however, in the so-called chronic abscesses, especially where the pus centers are small, as they frequently are when they originate in the hardened masses of exudation—generally remote from the surface—and, perhaps, surrounded by thickened walls, or further obscured by overlying structures. These conditions not only increase the difficulties of diagnosis, but the abscess is not as easily evacuated. It may be justifiable in doubtful cases, but where yet there is a strong suspicion of pus, to clear up the doubt by exploratory puncture; if pus is thereby revealed, the opening can be enlarged by divulsion with a steel dilator, the contents evacuated, the cavity antiseptically irrigated, and a drainage tube inserted. Care must, of course, be taken in the exploratory puncture, that the instrument penetrates deeply enough to reach through the thick abscess-wall, else there is liability to deception. The case is further complicated if there prove to be several small pus centers, instead of one, as is usual; but the rule applies to evacuate them all, if possible. If, under incision and drainage, the cavity does not close promptly, or if it secretes unhealthy pus, it should be washed out frequently with antiseptic solutions; or it may be packed with iodoform gauze. Finally, if it remain latent very long, and its pyogenic surface is converted into cicatricial membrane, the curette may be demanded before a cure is effected.

It may be stated, as a principle, that pus is here, as elsewhere in the body, an enemy for which diligent search should be made, unleashed when found, and the case further treated in accordance with the modern surgical rules applicable. It may be that the abscess can best be treated through the vagina, and the opening and drainage conducted through its walls. This will, most likely, be the case in low-down pus collections; but if it is impossible, or even very difficult, to reach the abscess through this route, then it may best be treated by abdominal section and drainage, and the careful stitching of the abscess walls to the margins of the incision. This class of inflammatory residues often tax the skill, patience, and resources of the gynecological surgeon as much as any pelvic disease, and it is one of the creditable achievements of his art that so much has been accomplished for their relief.

In the matting together of uterus, tubes, ovaries, and, possibly, intestines, by old inflammatory adhesions, very little can be done except through surgery. When abdominal section discloses these dire conditions, it may become a question sometimes whether the appendages shall be removed entire, or whether the adhesions shall only be broken up, according to the method of Dr. Wm. M. Polk, and the organs left in the hope that, though their functions may not be restored, the symptoms may be relieved. This latter method avoids that mutilation which so many dread, and may, in exceptional cases, serve as a valuable expedient.

Though the management of many other inflammatory products might be profitably discussed, I shall content myself, at this time, with but brief reference to one other class only, viz.: the residues resultant from salpingitis known as pyo- hydro- and hematosalpinx. This is a field of much interest, much debate, and much contest withal—one fraught with much difficulty, and one where there are yet many opinions and many methods. It is safe to say that more literature has appeared upon this subject and those akin to it within the last few years, than upon any other branch in gynecology—and the end is not yet. It is probable that a salpingitis may originate by extension from either the uterus or ovary, that it may begin as a catarrhal or a gonorrhœal inflammation, and that it may leave as residues either a dropsy of the tube or pyosalpinx, according to various surrounding and limiting circumstances. It is not always possible to differentiate these two conditions. The necessary bimanual manipulation to effect such diagnosis, involves the possible danger of rupture of the tube-wall, hence must be conducted with great care. These tube-sacs sometimes empty spontaneously through the uterus and out, in which event the diagnosis is no longer in perplexing doubt. The management, however, will not, up to this time, differ materially in either case. If the accumulations are small, we may seek to promote either their absorption or their exit through the uterus, by support and gentle elastic pressure through the agency of the wool tampon, by local resorbents, and by various constitutional means. If there are few and weak adhesions, and a coincidence of many other favorable circumstances, there is reasonable hope of accomplishing one or other of these terminations; the first in strong constitutions with good resisting powers; the second in feebler patients, where the uterine end of the tube is not too firmly sealed.

In the larger accumulations which threaten life, or greatly impair health, or even cause great bodily suffering, relief must come through surgical means. The danger of rupture or slow perforation of the sac is an ever-present one, to which due weight should be given in determining operative interference. In some cases, it may be advisable to resort to puncture of the tube-sac through the vagina. This method is available in small cyst-like tumors that are within easy reach. These sometimes remain unfilled after a single puncture, and the case proceeds to recovery. In other instances, repeated puncture becomes necessary, unless drainage is provided and antiseptic irrigations employed.

The question of removal of the appendages by abdominal section for various conditions that may be classed as inflammatory remnants, brings us into a field of great importance, and where, notwithstanding all that has been written and said of late, there is still room for much discussion. That the operation is a justifiable procedure in certain junctures—that it has a field—cannot be gainsaid. The principal and all-important question is to decide its applicability to each particular case. Are there no other means of cure? Shall the operation be done now, or shall there be further delay? These points are often thrust at the gynecological surgeon for decision, after other difficult problems have been disposed of and the way finally cleared for a judgment upon them. It is not here that a mere knowledge of the technique of the operation will avail, nor the courage to do it; but that rare combination of tact and skill which can only be found in a matured surgical judgment, is required for the exigency. Dr. Parvin, in a recent review (*American Journal of Medical Sciences*, February, 1888, p. 155), says: "A pressing question of the hour is, When is pelvic peritonitis a mere consequence usually of disease of tubes or ovaries, to be treated medically, and when is extirpation of the uterine appendages necessary? The latter question is not to be determined by the experience of one individual, no matter how great that is, but by that of numerous members of the profession, who shall wisely observe and carefully record not only immediate, but remote, results from the removal of the uterine appendages."

That the operation has been done when it ought not to have been done, is probably true. I have no doubt it is equally true that it has been left undone when it ought to have been done.

There are yet many opinions and many methods concerning the earlier conduct of these cases; but, given a tube distended with pus, no matter what its origin, if, after a reasonable trial of minor measures, it still renders existence miserable, torturing the patient and jeopardizing her life, there is, I affirm, no rational mode of relief excepting through the avenue of excision. I would not make haste to extirpate every swollen tube; I would exhaust other means first, and then, if need be, cut them out to save life or relieve grievous bodily suffering.

With the abdomen once open, various other questions are presented that must be decided, and that quickly. Shall the tube-sac be simply punctured and only the contents withdrawn? Shall the tube and ovary on one side only be removed? Shall the appendages on both sides be extirpated? Shall adhesions be broken up and the organs left? The operation may be complicated with other and new-found conditions, such as fibromata, metric, parametric, and peritoneal residues, cancerous and tubercular diseases, etc. If removal of a tube becomes impossible by reason of many and strong adhesions or friability of the tissues, we may then content ourselves with incision of the sac, sewing its edges to the abdominal wound, and drainage; or the abdominal opening may be sutured, and drainage through the vagina employed, according to Martin.

If the disease is wholly confined to one side, the other tube and ovary being perfectly healthy, then extirpation may be limited to the diseased side. Great circumspection will often be required to determine this question, as it frequently becomes a cause of regret, if subsequent mischief arises in the organs left behind, that they were not included in the first operation. It has been urged that the appendages should be saved on one side, unless there be very marked evidences of disease, lest the woman be sterilized by the operation. It is a burning question with me whether a woman who has had sufficient pelvic disease to warrant the unilateral extirpation of the appendages, should ever again be subjected to the dangers of pregnancy and parturition. In cases of doubt, I should lean to the side of total extirpation.

The decision might, with propriety, be left with the parties in greatest interest in each case, upon a full explanation beforehand.

It does not come within the province of this paper to treat upon the technique of the operation.

Many other details concerning the management of the residues of pelvic inflammations merit careful consideration, but this paper has already reached the limits which are proper in this paper.

In the foregoing, I have sought to underscore the following points, viz.:

1. The importance of early attention to even the *soi-disant* minor pelvic inflammations.

2. The suggestion of a simpler classification of pelvic inflammations.

3. The influence inflammatory remnants exert upon the health of women.

4. The great value, *inter alia*, of support and pressure in their management.

5. The thorough trial of all intermediate therapeusis before,

6. The final resort to surgical relief by abdominal section.

284 Franklin Street, June, 1888.

